# Experiences with Medinal-Schering

**Published:** 1909-04

**Authors:** 


### Experiences with Medinal-Schering

Dr. Ludwig Ebstein (*Muenchener med. Wochenschrift,* January 19, 1909, says: In the July number of *Therapie d. Gegenwart,* Ernst Steinitz of Berlin, recommended the monosodium salt of diethyl-barbituric acid as a substitute for diethyl-barbituric acid, which, under the name of veronal, is probably the most current hypnotic of today. The drawbacks of veronal,—the frequently slow appearance of sleep and the not rare aftereffects—are due to its relatively limited water solubility. The monosodium salt of the acid has the advantage of ready solubility; and, because of its more rapid absorption and speedier excretion, its effect is generally more prompt and certain than that of diethyl-barbituric acid in the same dose.

He details eighteen cases, in all of which medinal had a prompt and reliable effect without unpleasant by- or after-effects. All patients took it without trouble and tolerated it well. Not only as a hypnotic, but also for alleviation and abbreviation of stenocardic, asthmatic and the like conditions, medinal has done him good service. The observations in one case, with medinal as auxiliary in the treatment of morphine habit, may incite to further like trials.

The dominating characteristic of medinal above all other hypnotics, especially over diethyl-barbituric acid, is its diverse applicability. The water solubility of the remedy enables its employment per rectum in cases with sensitive stomach or vomiting, and in restlessness and excitability it may be given subcutaneously. Administered rectally it acts even more promptly than internally. The subcutaneous injection does not act so quickly as the rectal application, but is more intensive and protracted in effect.

